# Microscope-Assisted Coronary Artery Bypass Grafting: Technique and Results

**DOI:** 10.21470/1678-9741-2019-0416

**Published:** 2020

**Authors:** Andrey Semchenko, Alexander Makarov, Ilya Karpov, Mihail Zharenkov

**Affiliations:** 1 Federal Center for Cardiovascular Surgery of Healthcare Ministry of the Russian Federation, Khabarovsk, Russia.; 2 Far Eastern State Medical University of Healthcare Ministry of the Russian Federation, Khabarovsk, Russia.

**Keywords:** Coronary Artery Bypass, Myocardial Revascularization, Sutures, Anastomosis, Surgical

## Abstract

The microscope-assisted coronary artery bypass grafting (CABG) is a special technique of direct myocardial revascularization by the operating microscope using special equipment and atraumatic sutures. This method allows to complete elimination of technical errors during the performance of distal anastomoses and can be used to improve the outcomes and quality of conventional technique of operations. This article focuses on a detailed description of the technique for performing a distal anastomosis using a microsurgical technique and an operating microscope. Immediate results of operations are also reported. The data obtained suggest that microscope-assisted CABG is a safe, effective and reproducible procedure.

**Table:** 

Abbreviations, acronyms & symbols	
**CABG** **CPB** **ITA** **PCABG**	**= Coronary artery bypass grafting** **= Cardiopulmonary bypass** **= Internal mammary arteries** **= Pediatric microscope-assisted coronary bypass grafting**

## INTRODUCTION

Coronary artery bypass grafting (CABG) is one of the most effective and widely used surgical methods for the treatment of ischemic heart diseases. The results of operations depend on the patency of the coronary grafts. Coronary graft occlusion can lead to subsequent adverse cardiovascular events such as return of angina, repeat revascularization, myocardial infarction and death. It is known that long-term grafts patency is affected by the development of intimal hyperplasia and the progression of atherosclerosis. However, in the first days and weeks after surgery, grafts occlusion occurs due to thrombosis. The most frequent cause of early graft failure is technical defects in performing a distal anastomosis^[1-3]^. 

The microscope-assisted CABG is a special technique for direct myocardial revascularization by the operating microscope using special equipment and atraumatic sutures. This method allows the complete elimination of technical errors during distal anastomoses and can be used to improve the outcomes and quality of the conventional surgical technique^[4]^.

We have described the general principles of microscope-assisted CABG in previous publications. This article focuses on a detailed description of the technique for performing a distal anastomosis using a microsurgical technique and an operating microscope. Immediate results of operations are also reported.

### Description of the Technique

**Fig. 1:**
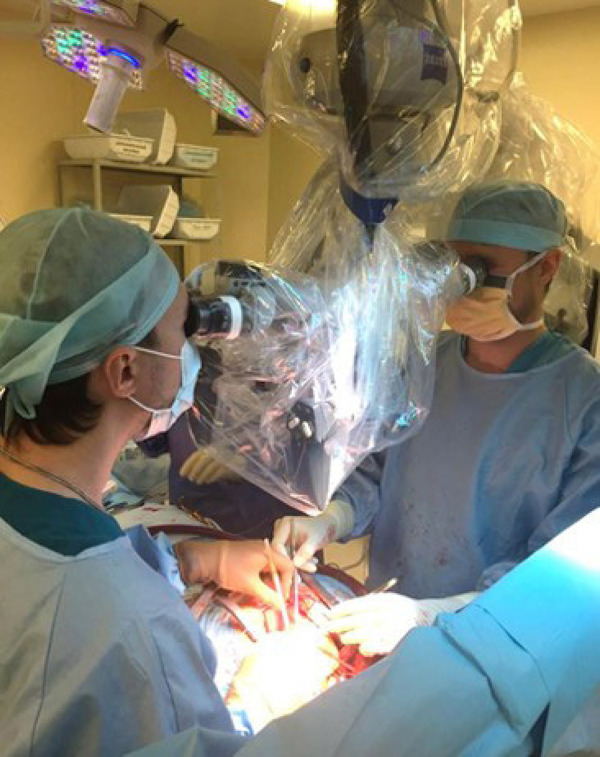
Microscope-assisted coronary artery bypass grafting.

**Fig. 2:**
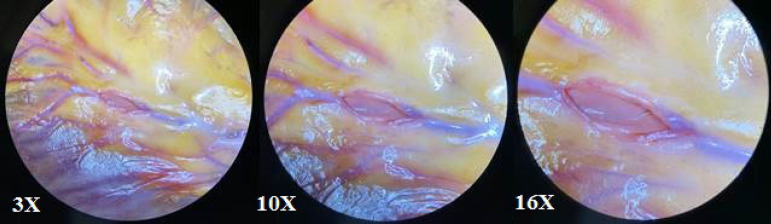
The same coronary arteriotomy in operating microscope field of view at different zooms.

Standard median sternotomy was used to approach the heart. All interventions were performed under normothermic cardiopulmonary bypass (CPB), with aortic cross-clamping and antegrade warm blood cardioplegia. The CPB was established by cannulation of the ascending aorta and the right atrium. The great saphenous vein and the left and right internal mammary arteries (ITA) were used as grafts. Adequate exposure of the corresponding cardiac surface was achieved before each distal anastomosis by enucleation of the heart and filling the oblique pericardial sinus with gauze. Use of apodactylic technique for distal anastomosis (all manipulations are done only with instruments, without contact with the surgeon's fingers), microsurgical sutures (8/0-9/0) and instruments, operating microscope (Zeiss OPMI Vario/S88) with magnification up to 24× was an important feature (Figures 1 and 2). All proximal anastomoses of the vein grafts were sewn every time into ascending aorta in the usual manner without assistance of an operating microscope. Both mammary arteries were applied *in situ.* The same surgeon performed all operations.

Distal anastomosis with microsurgical principles was performed as follows. The epicardium and then the arterial lumen were opened in the future anastomosis area using a scalpel. Arteriotomy was extended with angular and reverse scissors of 4 to 6 mm. Care should be taken to avoid injuring the opposite wall when performing the arteriotomy. The end of the conduit is cut obliquely and incised longitudinally. The size of the arteriotomy corresponds to the size of the conduit diameter. 

**Fig. 3:**
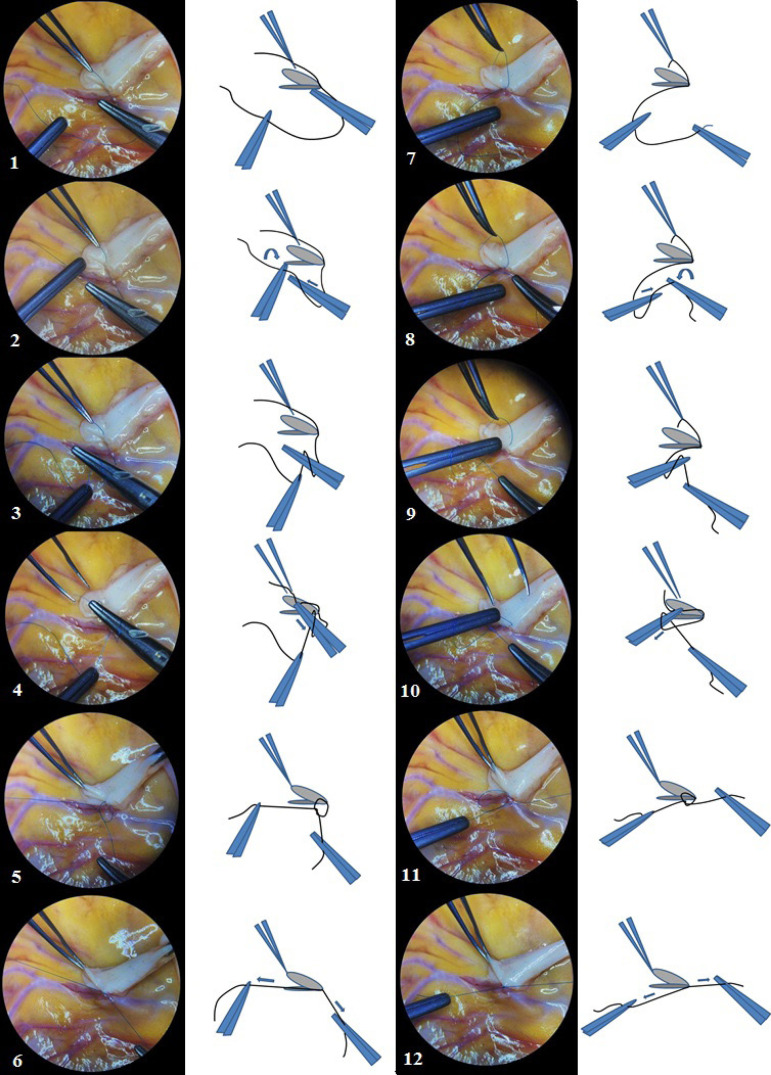
Microsurgical knot-tying technique by intercepting ends of the suture thread. Steps 1 to 6 – first knot, steps 7 to 12 – second knot. The third knot is tying in the same manner as the first knot (steps 1-6).

The first seam is performed by 8/0 (or 9/0) polypropylene suture with a length of about 10 cm and fixed by three ties at the “heel” of the anastomosis, as shown step by step in Figure 3. This knot-tying method is called a technique with intercepting ends of threads.

**Fig. 4:**
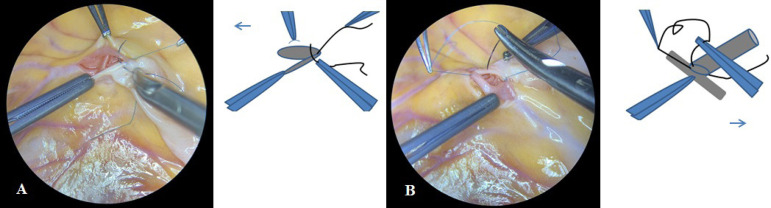
Suture is applied counterclockwise, first completing the far wall of the arteriotomy.

We continue to perform the anastomosis using microsurgical technique. The suture is placed in a continuous fashion, extending proximally from the heel to the toe. The far wall of the arteriotomy must be completed first. The direction of the suture in a clockwise or counterclockwise direction is determined by the position of the heel of the anastomosis. The suture is applied clockwise with injection of the needle from outside to inside through the arterial wall and from inside to outside through the conduit wall in case the heel of the anastomosis is directed towards the patient’s head. The suture is applied counterclockwise with injection of the needle from outside to inside through the conduit wall and from inside to outside through the arterial wall in case the heel of the anastomosis is directed towards the patient’s legs (Figure 4).

It is possible to use short-thread 8-0/9-0 polypropylene double-armed microsuture with a needle on both sides of the suture. In this case, the first two-thirds of the anastomosis are performed with one end of the suture thread starting from far wall of the arteriotomy in the same direction as for one-armed suture. The remaining one-third of the anastomosis is completed with another end of the suture thread.

**Fig. 5:**
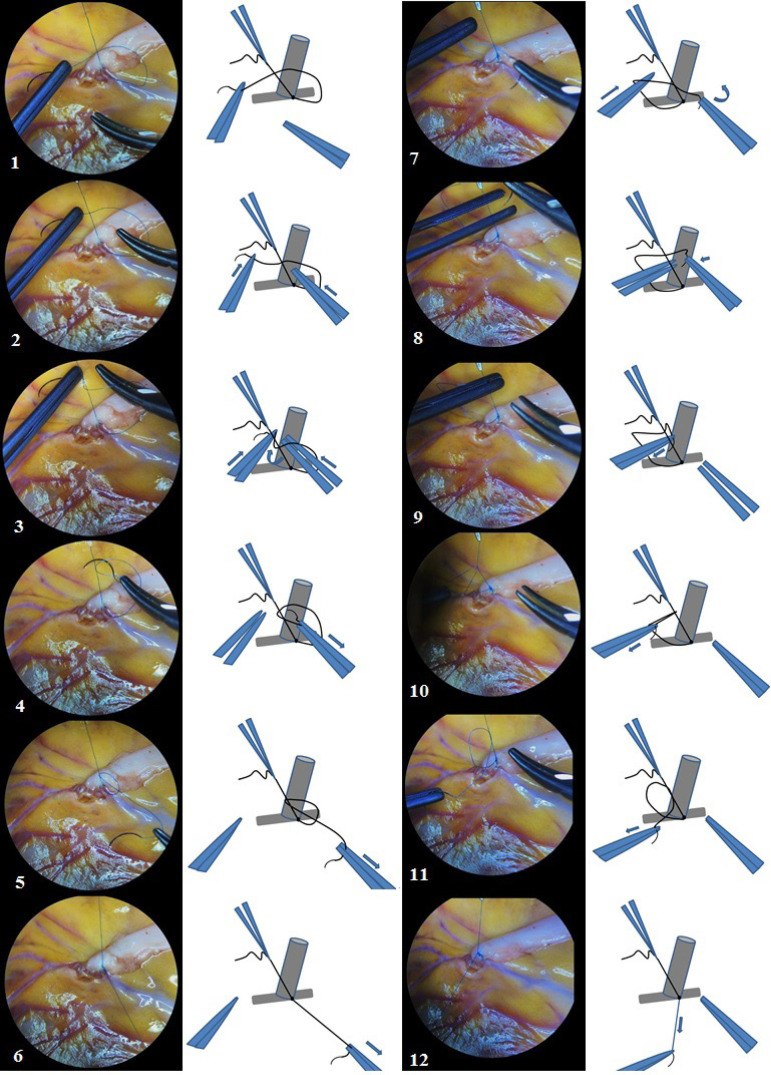
Microsurgical knot-tying technique with constant retention at one end of the suture thread. Steps 1 to 6 – first knot, steps 7 to 12 – second knot. The required number of knots tying in a same manner, successively repeating steps 1 to 12.

Anastomosis is finished by 6-8 ties with remaining end of the suture on the “heel” of the anastomosis, using the following microsurgical knot-tying technique with constant retention of one end of the suture thread as shown step by step in Figure 5. Every action was performed using microsurgical instruments and surgical microscope in a single visual field for surgeon and assistant under initial 10× magnification. Subsequent surgical stages did not have principal features.

## RESULTS

From October 2013 to October 2019, we performed 321 microscope-assisted CABG surgeries, of which 280 were isolated and 41 were associated with heart valve surgery (14 procedures), left ventricular aneurysm repair (12 procedures), surgical ablation for atrial fibrillation (10 procedures) or carotid endarterectomy (5 procedures). From 1 to 5 distal anastomoses were created intraoperatively. There were 1029 grafts, including 647 (62.9%) vein grafts and 382 (37.1%) ITA-grafts. Coronary endarterectomy was performed in only five cases. The revascularization index was 3.2. All grafts were patent according to intraoperative blood flow assessment by indocyanine green angiography with SPY imaging system (Novadaq Technologies Inc., Canada) and/or transit-time flowmetry (Medistim ASA, Norway) performed before chest closure. The mean myocardial ischemia and CPB times were 43.7±16.4 and 73.3±24 minutes, respectively. 

The in-hospital mortality rate was 1.9%. Postoperative angina was absent in all patients. In-hospital rates of acute cerebrovascular accident, myocardial infarction and repeated revascularization were 3.1%, 1.2% and 0.6%, respectively. All patients were discharged 13.9±4.5 days after surgery (Table 1). 

**Table 1:** Preoperative characteristics, intraoperative data and postoperative outcomes.

	Variables	No.	%
Preoperative characteristics	Number of patients	321	100
	Age (years)	62±7.2	
	Males	245	76.3
	Body mass index (kg/m^2^)	29.8±4.6	
	Angina class III or IV	171	53.3
	Previous myocardial infarction	189	58.9
	NYHA class III or IV	27	8.4
	Hypertension	304	94.7
	Diabetes	90	28
	Chronic obstructive pulmonary disease	80	24.9
	Smoking history	119	37.1
	Chronic kidney disease	42	13.1
	Stroke	39	12.1
	Previous percutaneous coronary intervention	66	20.6
	Multifocal atherosclerosis	58	18.1
	Left ventricular ejection fraction (%)	59.7±9.6	
Preoperative angiographic data	Left main trunk disease	81	25.2
	1 vessel	9	2.8
	2 vessels	58	18.1
	3 vessels	254	79.1
Intraoperative data	Isolated CABG	280	87.2
	CABG + mitral valve repair	6	1.9
	CABG + mitral valve replacement	2	0.6
	CABG + aortic valve surgery	5	1.6
	CABG + tricuspid valve repair	1	0.3
	CABG + surgical ablation for atrial fibrillation	10	3.1
	CABG + left ventricular aneurysm repair	12	3.7
	CABG + carotid endarterectomy	5	1.6
	Single ITA	242	75.4
	Bilateral ITA	65	20.2
	SVG use	306	95.3
	Coronary endarterectomy	5	1.6
	Number of grafts		
	1	9	2.8
	2	45	14
	3	151	47
	4	103	32.1
	5	13	4.1
	Revascularization index	3.2	
	Complete revascularization	283	88.2
	Time of myocardial ischemia (min)	43.7±16.4	
	CPB time (min)	73.3±24	
	Operation time (min)	184.7±41.4	
Postoperative outcomes	In-hospital mortality	6	1.9
	Acute cerebrovascular accident	10	3.1
	Myocardial infarction	4	1.2
	Repeated revascularization	2	0.6
	Atrial fibrillation	74	23.1
	Acute kidney failure	1	0.3
	Respiratory complication	9	2.8
	Bleeding	8	2.5
	Mediastinitis	3	0.9
	Hospital stay (days)	13.9±4.5	

Among the patients studied, 259 (80.7%) underwent multislice computed tomography angiography after operation before hospital discharge. The patency of 835 coronary bypass grafts (526 venous and 309 mammary) was evaluated. There were 95.8% (504/526) patent autovenous grafts and 95.1% (294/309) patent ITA-grafts. 

## DISCUSSION

The operating microscope and the microsurgical technique for direct myocardial revascularization were first applied by G. Green in 1968 (USA) in cardiovascular surgery^[5]^. Series reports regarding the use of microsurgical techniques for CABG were made in different years by P. Jairaj (Australia), D. Loisance (France), K. Katsumoto (Japan), R. Akchurin (Russia), J. Catapano (Canada) and S. Spagnolo (Italy)^[6-11]^.

The microscope-assisted CABG has several advantages over the conventional method. The double optical control of the operator and the assistant, the use of microsurgical suture materials and optimal visualization in microscope-assisted CABG make it possible to achieve high-precision coronary anastomosis, performed in any condition of the distal coronary bed. The common field of view of the operator and assistant when working with an operating microscope, and the ability to adequately correct the magnification according the diameter of the target coronary artery allow to identify and prevent in time several most common technical errors in the execution of a distal anastomosis, such as the capture of opposite walls in the suture, fragmentation of the atherosclerotic plaque with its screwing in the lumen, trauma to the posterior wall of the coronary artery, etc.^[4,8,12]^.

It is well known that continuous suture is widely used in coronary surgery. Several researchers have shown that this type of suture is potentially dangerous regarding the development of narrowing of small diameter vessels at the anastomotic site. The “purse-string” effect due to uncontrolled tightening of the suture, excessive capture of surrounding tissues in the suture and additional hemostatic sutures can lead to significant stenosis of the anastomosis between the conduit and the coronary artery. These unsatisfactory effects can be prevented by using microsurgical technique and operative microscope. Microsuture (8/0-9/0) has less influence on the thickness of the vascular suture. A significant optical magnification makes it possible to compare vessel walls very accurately, without the need for additional sutures. It significantly reduces the risk of developing hemodynamically significant stenosis along the anastomosis line^[4,6,11]^.

The use of the surgical microscope for CABG allows the restoration of blood flow in the coronary arteries with a diameter of less than 1.5 mm. Enough magnification and microsurgical technique allow complete myocardial revascularization in patients with a small diameter of the coronary arteries and reduce the rate of refusals to operate for this reason^[4,7,9,13]^.

It was determined that the use of operating microscope is an independent predictor of survival after CABG, both in the early and long-term postoperative period^[4,9]^. According to the recent study, the use of microsurgery provides the best clinical results in coronary surgery afterwards instead of the traditional technique. It was shown that the 10-year survival in the case of the microsurgical method (n=89) compared to the conventional CABG technique (n=104) was 84.3 and 70.2% (*P*=0.03); the incidence of non-fatal myocardial infarction was 9.6 and 21.2% (*P*=0.03); the angina recurrence was 14.4 and 25.5% (*P*=ns); and the reoperation rate was 10.8 and 22.3% (*P*=0.04), respectively. It is noteworthy that, in this study, the number of anastomoses in arteries of less than 1.5 mm was significantly higher in the microsurgical group - 178 *vs.* 86 (*P*≤0.001), respectively^[14]^.

The microscope-assisted pediatric coronary bypass grafting (PCABG) for congenital heart diseases is also developing in recent years. Due to the rare, but serious, early or late occurrence of ischemic myocardial complications after radical operations that require a coronary transfer, the importance of PCABG for children has gradually increased. Despite saphenous vein grafts have been used in the past, the use of ITA has been widely adopted and is now used more frequently due to documented angiographic and clinical superiority, with improved long-term patency rates and growth potential. Small children’s ITA is a short, thin-walled artery with a diameter of 1 mm or less. Thus, coronary anastomosis with ITA for children requires special technical solutions and microsurgical techniques, including special instruments, 8-0 or 9-0 microsuture material, and reconstruction by operating microscope^[10,15]^.

One of the reasons preventing the popularization of microscope-assisted CABG is the need to equip operating rooms with operating microscopes. It is associated with financial costs. But the economic efficiency of microscope-assisted CABG in the long term has not yet been analyzed. However, an operating microscope is used in many fields of surgery. This is common for surgeons who perform neurological, gynecological, ophthalmologic, otorhinolaryngological (middle ear) interventions, plastic surgery, etc. As a rule, multidisciplinary surgical clinics are equipped with operating microscope. This equipment can also be used in the cardiovascular operating room. Future investigations will confirm or refute the economic justification for individual equipment.

There is no doubt that the use of operating microscope and the microsurgical technique improve the quality of the anastomosis performed during CABG. But this is not the only factor in the subsequent grafts’ patency. Despite the fact that the patency of all grafts was confirmed by indocyanine green angiography and/or transit-time flowmetry intraoperatively, we found that about 5% of all grafts undergo early occlusion, regardless of their type. Therefore, further studies are needed to define the causes of coronary grafts failure in early postoperative period after CABG.

**Table:** 

Authors' roles & responsibilities	
AS	Substantial contributions to the conception or design of the work; or the acquisition, analysis, or interpretation of data for the work; drafting the work or revising it critically for important intellectual content; agreement to be accountable for all aspects of the work in ensuring that questions related to the accuracy or integrity of any part of the work are appropriately investigated and resolved; final approval of the version to be published
AM	Substantial contributions to the conception or design of the work; or the acquisition, analysis, or interpretation of data for the work; drafting the work or revising it critically for important intellectual content; agreement to be accountable for all aspects of the work in ensuring that questions related to the accuracy or integrity of any part of the work are appropriately investigated and resolved; final approval of the version to be published
IK	Substantial contributions to the conception or design of the work; or the acquisition, analysis, or interpretation of data for the work; drafting the work or revising it critically for important intellectual content; agreement to be accountable for all aspects of the work in ensuring that questions related to the accuracy or integrity of any part of the work are appropriately investigated and resolved; final approval of the version to be published
MZ	Substantial contributions to the conception or design of the work; or the acquisition, analysis, or interpretation of data for the work; drafting the work or revising it critically for important intellectual content; agreement to be accountable for all aspects of the work in ensuring that questions related to the accuracy or integrity of any part of the work are appropriately investigated and resolved; final approval of the version to be published
